# Case report: A rare manifestation of vasospasm induced myocardial infarction with ST-segment elevation in a young male patient

**DOI:** 10.3389/fcvm.2022.1017107

**Published:** 2023-01-12

**Authors:** Laurynas Diečkus, Greta Rodevič, Arvydas Baranauskas, Giedrius Davidavičius, Povilas Budrys

**Affiliations:** ^1^Clinic of Cardiac and Vascular Diseases, Faculty of Medicine, Vilnius University, Vilnius, Lithuania; ^2^Center of Internal Diseases, Vilnius University Hospital Santaros Clinics, Vilnius, Lithuania; ^3^Cardiology and Angiology Center, Vilnius University Hospital Santaros Klinikos, Vilnius, Lithuania

**Keywords:** STEMI, vasospasm, young patient, CAS, case report

## Abstract

**Background:**

Minority of acute myocardial infarctions (MI) are caused by a non-atherosclerotic occlusion of the coronary artery. We present a case report, where MI with ST-segment elevation was provoked by a vasospasm, which is a rare aetiological finding.

**Case presentation:**

27-year-old male patient presented to the emergency department because of a sudden onset chest pain radiating to the left arm. The patient underwent percutaneous coronary intervention (PCI) to the right coronary artery (RCA) 3 months ago due to inferior wall MI, however, chest pain episodes kept on recurring at night throughout the whole period after the intervention. During current admission, initial electrocardiogram (ECG) demonstrated ST-segment elevation in leads II, III and aVF. Coronary angiogram revealed diffuse severe narrowing of the right coronary artery, which was relieved with intracoronary administration of nitrates and verapamil. After coronary angiogram patient was given oral long-acting nitrates and verapamil, however, during the following days nocturnal chest pain episodes reoccurred. It was decided to swap verapamil to diltiazem, which led to complete cessation of angina episodes. The patient was discharged in stable condition and symptom free. It was suspected that the first MI was of vasospastic origin, which likely led to unnecessary stenting.

**Conclusions:**

This clinical case has demonstrated the challenges clinician could face in order to correctly diagnose vasospasm-induced MI because of its rare occurrence and highly variable presentation. We strongly suggest using intracoronary nitroglycerine during coronary angiography as a standard practice to avoid a potential diagnostic error and unnecessary stenting. Although, in some cases the reason behind coronary artery spasm (CAS) remains unclear, medical treatment can be very effective for CAS prevention.

## Introduction

Myocardial infarction with ST-segment elevation (STEMI) is an event during which myocardial ischemia results in myocardial injury or necrosis (1). The dominant cause for this emergency condition is acute atherosclerotic plaque rupture followed by a total coronary artery occlusion (2). However, it is estimated that in 3–25% of all acute MI cases the cause is of non-atherosclerotic occlusion origin, one of which is vasospasm (3–5). Women have 5-times higher chance of presenting with MI without a ruptured atherosclerotic plaque than men; the incidence is also higher in non-Caucasian patient group (6). In this case report we present a young Caucasian male patient who had inferior wall STEMI caused by profound vasospasm in the right coronary artery (RCA).

## Case description

**Figure 1:**
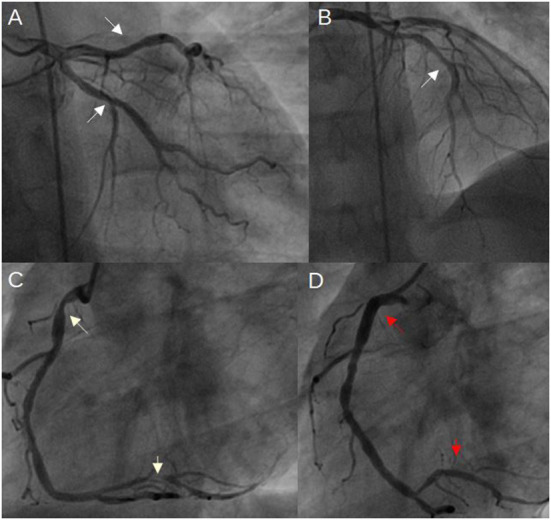
Coronary angiogram 3 months ago, when inferior wall MI occurred. **(A, B)** mild coronary artery narrowings in both LCx and LAD (white arrows); **(C)** severe narrowings in the RCA (white arrows); **(D)** RCA after stents implantation (red arrows indicate both implanted stents) (LAD, left anterior descending artery; LCx, left circumflex artery; MI, myocardial infarction; RCA, right coronary artery).

**Figure 2:**
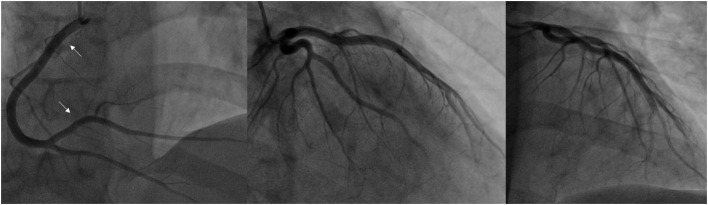
Coronary angiogram when recurrent chest pain episodes occurred after PCI. Both left and right coronary arteries are patent, their filling is unimpaired. No narrowings in the mid RCA, LAD and LCx, which were observed during the initial coronary angiogram, are present. White arrows indicate stents implanted in the RCA (LAD, left anterior descending artery; LCx, left circumflex artery; RCA, right coronary artery).

27-year-old male patient presented to the emergency room because of a sudden onset severe chest pain (8-9 points on visual analog scale) radiating to the left arm and neck. Three months ago he was admitted to a different percutaneous coronary intervention (PCI) center due to inferior wall STEMI and two drug-eluting stents were implanted: one in the proximal part of the RCA, the other in the right posterolateral (rPL) branch (Figure 1). During the one-month period after procedure the patient complained of recurrent episodes of chest pain at rest, not related to exertion, similar to those experienced during the heart attack but less intense. In the same PCI center a repeat coronary angiogram was performed due to a suspicion of unstable angina but no visual stenoses were identified, coronary artery flow was not impaired (Figure 2, Table 1). Patient was advised to continue dual antiplatelet therapy and atorvastatin. A week prior to current admission, almost every night he had episodes of chest pain lasting 15–30 min and ceasing spontaneously. Past medical history included hypertension, dyslipidemia, smoking, excessive alcohol consumption prior to the first episode of STEMI and COVID-19 infection 9 months ago with no residual symptoms. He was not suffering from any other chronic disease.

**Table 1:** Timeline of the clinical case.

**Date**	**Event**
Three months prior to current admission	Inferior wall STEMI, two drug-eluting stents placed.
Two months prior to current admission	Due to recurring chest pain episodes repeat coronary angiogram was performed, no stenoses identified.
One week prior to current admission	Every night patient experienced chest pain episodes, which ceased spontaneously.
Day of admission (day 1)	Patient presented to ER because of a sudden onset severe chest pain radiating to the left arm and neck. ECG showed ST elevations in inferior wall leads, patient was taken for an urgent coronary angiogram. It revealed diffuse severe and mild narrowings of coronary arteries which were successfully treated with intracoronary injections of nitroglycerine and verapamil. Chest pain stopped.
Day 3	During the night, patient complained of chest pain which subdued after taking nitroglycerine.
Day 6	Nocturnal chest pain episodes persisted despite increasing verapamil dosage–medication was switched to diltiazem.
Day 13	After changing medication, no more chest pain episodes occurred. Patient was discharged in stable condition.

ECG, electrocardiogram; ER, emergency room; STEMI, ST-elevation myocardial infarction.

## Diagnostic assessment

**Figure 3:**
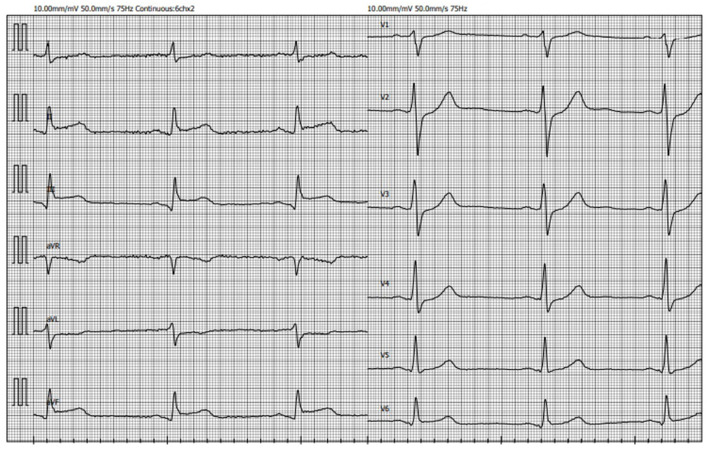
ECG recorded on admission demonstrating ST-segment elevation in leads II, III, aVF with reciprocal ST-segment depression in leads I and aVL (ECG, electrocardiogram).

**Figure 4:**
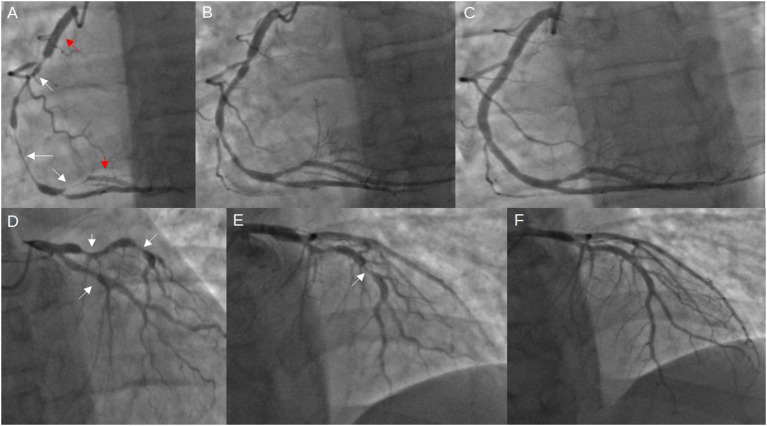
Coronary angiogram from the current stay. **(A)** severe diffuse RCA spasm (indicated by the white arrows), previously implanted stents (red arrows); **(B)** improved RCA spasm after intracoronary injection of 300 mcg nitroglycerine; **(C)** further resolution of vasospasm after administering additional 100 mcg nitroglycerine and 2.5 mg verapamil; **(D, E)** visible vasospasms in both LCx and LAD; **(F)** vasospasm resolution after 100 mcg nitroglycerine and 2,5 mg verapamil intracoronarily (LAD, left anterior descending artery; LCx, left circumflex artery; RCA, right coronary artery).

**Figure 5:**
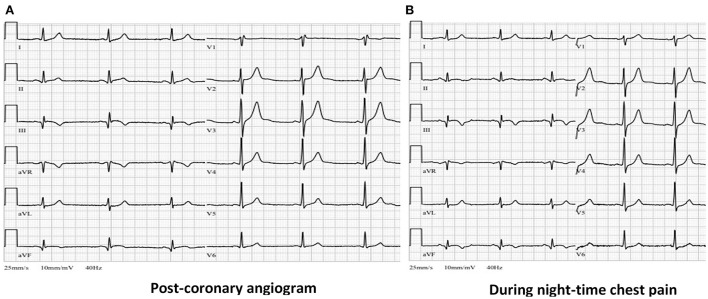
**(A)** Post-procedural ECG from ICU showing resolution of ST-segment elevation in leads II, III and aVF with formation of negative “reperfusion” T waves in III and aVF leads. **(B)** ECG during the night chest pain episode showing no significant changes compared with **(A)** (ECG, electrocardiogram, ICU, intense care unit).

During the current hospital admission, an initial electrocardiogram (ECG) showed ST-segment elevation in leads II, III and aVF with reciprocal ST-segment depression in leads I and aVL (Figure 3). Based on patient's complaints and ECG findings, inferior wall ST-elevation myocardial infarction (STEMI) was suspected and the patient was taken for an urgent coronary angiogram. It revealed a diffuse severe narrowing of the right coronary artery (RCA) and mild to moderate stenoses in both left anterior descending (LAD) and left circumflex (LCx) arteries (Figure 4). Suspecting vasospasm, the right coronary artery was injected intracoronarily with 300 mcg of nitroglycerine, which only partially relieved the spasm. Therefore additional 100 mcg of nitroglycerine and 2.5 mg of verapamil were administered and the luminal patency was successfully restored. The same drug combination was then injected into the left coronary artery (LCA) relieving its spasms as well. After intracoronary injections of medications chest pain stopped. It was confirmed that the inferior STEMI was secondary to a vasospasm in RCA. Post-procedural ECG showed the return of ST-segment to the baseline (Figure 5A). The patient was given ticagrelor 90 mg bd, aspirin 100 mg od, atorvastatin 80 mg od, omeprasol 20 mg od, amplodipine 5 mg bd, and verapamil 80 mg od. After 24 h, with no recurrent chest pain and in stable condition, patient was transferred out from the CCU to the regular cardiology ward.

Detailed medical examination and various tests were done to detect potential cause of vasospasm. The patient was consulted by a rheumatologist. However, no symptoms in patient's history related to vasculites or collagenoses were found and physical examination showed no abnormalities. Electrolyte panel, markers of inflammation, thyroid function tests, level of eosinophils in blood, markers of autoimmune diseases were within normal limits, drug abuse screening test came back negative as well (Table 2). Temporal artery duplex ultrasonography did not reveal any pathological findings either. Transthoracic echocardiogram was unremarkable with preserved left and right ventricle ejection fraction.

**Table 2:** Laboratory tests.

**Laboratory test**	**Initial values**	**Repeated values**	Reference values^*^
Inflammation markers (CRP, mg/l)	11.9	–	≤ 5
Electrolyte panel (mmol/l): • Potassium (K) • Sodium (Na) • Chloride (Cl)	K −4.2	K −4.4	K 3.8 −5.3
	Na −141	Na −138	Na −134-145
	Cl −106	Cl −104	Cl −98-107
Thyroid function test (TSH, mU/l; FT4, pmol/l; ATPO kU/l; T3 nmol/l)	TSH −1.323;	–	TSH −0.4-4,0
	FT4 −14.62;		FT4 −9.0-19.0
	ATPO−0.6		ATPO < 5.61
	T3 −1.52		T3 −0.89 −2.44
Drug abuse screening test (from urine)	Negative ()	–	Negative
Auto-immune disease markers	- Jo-1: Negative, - anti-beta2-GP1 Ig GAM: Negative, - DFS70: Negative, - dsDNR: Negative, - CENP-B: Negative, - AMA-M2: Negative, - SS-A: Negative, - Sm: Negative, - PM-Scl: Negative, ENA: - nRNP/Sm Negative, - PCNA: Negative, - Scl-70: Negative, - rib. P.-protein: Negative, - Ro-52: Negative, - nucleosomes: Negative, - SS-B: Negative, - histones: Negative.	–	Negative
Level of eosinophils in blood (^*^10^9^/l)	0.8	0.6; 0,7	0–0.7
Troponin I (ng/l)	10716	1047	≤ 35
Lipid panel (mmol/l)	Total cholesterol −2,21	–	Total cholesterol- < 5,2
	Triglycerides −0,87		Triglycerides– ≤ 1,8
	HDL-cholesterol −0,90		HDL-cholesterol->0,91
	LDL-cholesterol – 0,91		LDL-cholesterol- ≤ 3,0
Liver enzymes (U/L)	AST −329		AST– ≤ 40
	ALT −66		ALT- ≤ 40

* Reference values adapted from the laboratory of medical center patient was currently treated in.

ATPO, anti-thyroid proxidase; CRP, C-reactive protein; FT4, free thyroxine; T3, triiodothyronine; TSH, thyroid-stimulating hormone; HDL, high-density lipoprotein; LDL, low-density lipoprotein; AST, aspartate aminotransferase; ALT, alanine transaminase.

During the night of the third hospitalization day patient complained of chest pain, which subdued after taking nitroglycerine. The ECG recorded during the pain episode did not reveal any new electrocardiographic signs of myocardial ischemia (Figure 5B). Verapamil dosage was increased to 80 mg bd and isosorbide dinitrate (ISDN) 20 mg od was given as well. Whereas chest pain episodes persisted even after further increasing verapamil dosage to 80 mg tds, it was decided to switch verapamil to diltiazem. The optimal effect was achieved with a total daily dose of 360 mg diltiazem. No more chest pain episodes as well as potentially lethal rhythm disturbances were recorded.

The patient was discharged in stable condition without chest pain on the 13 day after arriving to the emergency room. He was prescribed ticagrelor for the next 10 months, aspirin, esomeprasol, statin, ISDN and diltiazem.

## Discussion

The reported case demonstrates a young male patient who had a vasospasm-induced inferior wall STEMI and had two stents implanted because previous MI was thought to be caused by a ruptured atherosclerotic plaque. Despite the treatment, chest pain episodes continued and only the third coronary angiogram led to the discovery that these angina attacks were of vasospastic origin. As seen in the angiographic images above, the spasms presented both as local and diffuse narrowings, which made it easy to be mistaken and be treated by implanting stents. The main cause of observed vasospasms remained unclear in this case.

Development of coronary artery spasm (CAS) depends on a wide range of risk and precipitating factors. Hung et al. listed older age, smoking and higher blood levels of C-reactive protein (CRP) as risk factors associated with increased susceptibility to developing CAS. In our case the patient was young and had normal CRP blood levels, however, he was a smoker. Smoking is associated with endothelial damage due to increased amount of reactive oxygen species (7). We can suppose that smoking could have been a contributing factor for spasms to occur. Furthermore, in the same article by Hung et al., mental/physical stress, parasympatho- and sympathomimetics, central nervous system (CNS) stimulants and cold pressor as a sympathetic activator were referred to as precipitating factors potentially contributing to the onset of CAS (8). The presented patient denied feeling stressed out mentally or physically. The patient denied the abuse of CNS stimulants or narcotics as well and it was confirmed with a negative drug abuse screening test.

CAS usually occurs to postmenopausal women and middle- or old-aged men. It is very uncommon for young men to develop CAS. Therefore, a genetic predisposition plays an important role in the pathogenesis of a coronary spasm. Genetic epidemiological studies have reported that several genetic variants of the genes for angiotensin-converting enzyme, angiotensin II receptor type, endothelial nitric oxide synthase (e-NOS) and paraoxonase are associated with the higher risk for coronary artery spasm (9–11). For example, several studies have discovered the polymorphisms of Glu298Asp in the exon 7 and T−786C in the 5′-flanking region of the e-NOS gene and have shown that these polymorphisms increase the risk of CAS (12, 13). However, genetic testing was not performed to the reported patient.

Triptans used to treat migraine are also known as triggers of coronary artery spasm through an action at 5-HT1B-receptors, which can be found in the coronary arteries (14–16). Because of vasoconstrictive activity triptans are contraindicated in cases of uncontrolled arterial hypertension or coronary artery disease (17). In our reported case the patient denied suffering from migraine and the use of triptans as well. Circadian variation was proposed in a work by Yasue et al. as another possible spasmogenic risk factor along the other ones which have already been mentioned. This risk factor may be relevant to our patient, since he complained of chest pain episodes during night rest, which is usual time for CAS to occur due to yet unelucidated pathological pathway (18). Moreover, Lee et al. demonstrated that subclinical hypothyroidism could be associated with coronary spasms (19). However, the thyroid function of our patient was unimpaired. In some rare cases eosinophilic granulomatosis with polyangiitis, formerly known as Churg-Strauss Syndrome, may also cause coronary spasms, which are believed to be mediated by vasoactive compounds produced by eosinophils (20). Considering this risk factor, the patient was consulted by the rheumatologist and various markers for auto-immune diseases were tested but they all came back negative. Rarely vasospasms could also be induced in the setting of an allergic or anaphylactic reaction–such condition is known as the Kounis Syndrome (KS). It describes an acute coronary syndrome, which is associated with mast cell and platelet activation during allergic reaction (21). The presented patient, however, had no symptoms of allergic reaction and blood level of eosinophils was normal every time it was checked during this hospital stay, thus KS as a possible cause of spasms was ruled out.

During CAS, various electrocardiographic findings can be present: ECG may be normal at the beginning of an attack or when the attack is mild, or ST-segment elevation/ depression may be visible. Furthermore, patients experiencing coronary spasms may present with or without symptoms, thus the diagnosis of CAS rarely can be made by symptoms or ECG findings alone (22). The reported patient had typical clinical and electrocardiographic presentation of STEMI with chest pain and ST-segment elevation on the ECG as well.

Provocation testing adjunctive to coronary angiography is considered to be the most reliable method for diagnosing CAS in cases when spasms are suspected to be the cause of angina episodes and need to be confirmed (23). Most commonly, intracoronary injection of ergonovine or acetylcholine are used to provoke coronary spasm (22). After the first episode of STEMI our patient had repeat angina attacks at rest, strongly suggestive of vasospastic angina. Therefore, second coronary angiogram was performed at the same hospital, which demonstrated completely normal coronary arteries. However, provocation testing for CAS was not done at that time, likely due to not enough experience in such testing. We could speculate that should the CAS provocation test was performed, the correct diagnosis could have been established earlier and with adequate medical treatment, the second MI could have been prevented. Thus, provocation testing or at least a trial of medical therapy should be encouraged when there is a high clinical suspicion of vasospastic angina. The third coronary angiogram, which was performed in a different (our) hospital, demonstrated various levels of coronary narrowings, which were relieved by the intracoronary injection of nitroglycerine and verapamil, confirming the spasm as a cause of angina.

The patient received long acting nitrates with verapamil at first, which was later changed to diltiazem due to recurring chest pain episodes. Both these medications are calcium channel blockers (CCB), which are effective for the prevention of vasospasms and angina attacks (18). As for the acute angina attacks due to CAS, sublingual nitrate remains the main treatment (23).

During the previous hospital stay our patient had stents implanted suspecting STEMI to be of atherosclerotic plaque rupture rather than of vasospastic origin. Intravascular imaging, such as intravascular ultrasound or optical coherence tomography, was not performed. We believe, that intravascular imaging should be strongly considered in clinical cases like this–when there are factors, associated with a high probability of a non-atherosclerotic MI, such as very young patient's age. In the study conducted by Katoh et al., it was discovered that in both infarct-related and unrelated arteries coronary spasms could be provoked by high frequency and that spasms after stenting were more likely to occur distally from the implanted stent (24). Similarly, diffuse coronary spasm was observed distal to the proximal stent in our patient. The authors recommend using CCB for suppressing further coronary spasms.

This clinical case has demonstrated the challenges one could face in order to correctly diagnose vasospasm-induced MI because of its rare occurrence and highly variable presentation. Though ACS caused by vasospasm is relatively rare, we strongly suggest using intracoronary nitroglycerine during coronary angiography as a standard practice to avoid a potential diagnostic error and unnecessary stenting. The recommended treatment for further CAS prevention is CCB, however, as seen in this case, it is also crucial to select the correct medication. Although, in some cases the reason behind CAS remains unclear, medical treatment can be very effective for CAS prevention as it was showed in this clinical case presentation.

## Data availability statement

The original contributions presented in the study are included in the article/supplementary material, further inquiries can be directed to the corresponding author.

## Ethics statement

Written informed consent was obtained from the individual(s) for the publication of any potentially identifiable images or data included in this article.

## Author contributions

LD and GR: conception and drafting of the work, acquisition, and interpretation of data. GD and AB: conception of and revising the work. PB: conception and drafting of the work and revising the work. All authors read and approved the final manuscript, as well as have agreed both to be personally accountable for their contributions and to ensure that questions related to the accuracy or integrity of any part of the work, even ones in which the author was not personally involved, are appropriately investigated, resolved, and the resolution documented in the literature.
